# Holidays, celebrations, and commiserations: measuring drinking during feasting and fasting to improve national and individual estimates of alcohol consumption

**DOI:** 10.1186/s12916-015-0337-0

**Published:** 2015-05-22

**Authors:** Mark A Bellis, Karen Hughes, Lisa Jones, Michela Morleo, James Nicholls, Ellie McCoy, Jane Webster, Harry Sumnall

**Affiliations:** College of Health and Behavioural Sciences, Bangor University, Bangor, LL57 2PZ UK; Public Health Wales, Hadyn Ellis Building, Cardiff University, Maindy Road, Cardiff, CF24 4HQ UK; Centre for Public Health, Liverpool John Moores University, 15-21 Webster Street, Liverpool, L3 2ET UK; Centre for History in Public Health, London School of Hygiene and Tropical Medicine, 15-17 Tavistock Place, London, WC1H 9SH UK

**Keywords:** Abstinence, Alcohol, Binge drinking, Holidays, Sales, Surveys

## Abstract

**Background:**

Accurate measures of alcohol consumption are critical in assessing health harms caused by alcohol. In many countries, there are large discrepancies between survey-based measures of consumption and those based on alcohol sales. In England, surveys measuring typical alcohol consumption account for only around 60% of alcohol sold. Here, using a national survey, we measure both typical drinking and atypical/special occasion drinking (i.e., feasting and fasting) in order to develop more complete measures of alcohol consumption.

**Methods:**

A national random probability telephone survey was implemented (May 2013 to April 2014). Inclusion criteria were resident in England and aged 16 years or over. Respondents (n = 6,085) provided information on typical drinking (amounts per day, drinking frequency) and changes in consumption associated with routine atypical days (e.g., Friday nights) and special dinking periods (e.g., holidays) and events (e.g., weddings). Generalized linear modelling was used to identify additional alcohol consumption associated with atypical/special occasion drinking by age, sex, and typical drinking level.

**Results:**

Accounting for atypical/special occasion drinking added more than 120 million UK units of alcohol/week (~12 million bottles of wine) to population alcohol consumption in England. The greatest impact was seen among 25- to 34-year-olds with the highest typical consumption, where atypical/special occasions added approximately 18 units/week (144 g) for both sexes. Those reporting the lowest typical consumption (≤1 unit/week) showed large relative increases in consumption (209.3%) with most drinking associated with special occasions. In some demographics, adjusting for special occasions resulted in overall reductions in annual consumption (e.g., females, 65 to 74 years in the highest typical drinking category).

**Conclusions:**

Typical drinking alone can be a poor proxy for actual alcohol consumption. Accounting for atypical/special occasion drinking fills 41.6% of the gap between surveyed consumption and national sales in England. These additional units are inevitably linked to increases in lifetime risk of alcohol-related disease and injury, particularly as special occasions often constitute heavy drinking episodes. Better population measures of celebratory, festival, and holiday drinking are required in national surveys in order to adequately measure both alcohol consumption and the health harms associated with special occasion drinking.

**Electronic supplementary material:**

The online version of this article (doi:10.1186/s12916-015-0337-0) contains supplementary material, which is available to authorized users.

## Background

Alcohol is related to over 200 different health conditions with recent estimates suggesting it was responsible for 5.1% of the global burden of disease and injury (disability adjusted life years) and 3.3 million deaths in 2012 [1]. Internationally, there is a strong positive relationship between average alcohol consumption per capita and reported levels of alcohol-related disease and mortality, both across countries and within countries over time [2,3]. At an individual level, the vast majority of conditions caused entirely by alcohol (e.g., alcohol-related liver disease) or partly attributable to alcohol (e.g., breast cancer) show increases in risk with increasing levels of personal consumption [4]. However, a few show J- or U-shaped relationships with potential, yet contested, health benefits at low levels of consumption [5-7]. Consequently, from a public health perspective, both national trends in average alcohol consumption and how much consumption is at health-harming levels are important considerations when examining the impacts of alcohol on health.

Typically, data on trends and demographic variation in alcohol consumption are provided by large-scale surveys and records of amounts of alcohol products sold for consumption [8]. Thus, in England, demographic information is available from the Health Survey for England (HSE [9]), while taxation information is also used as a proxy for sales [10,11]. However, in England and many other countries with both data sources, national estimates of alcohol consumption based on survey data account for only a fraction of the total sold [1]. Thus, a UK study found that survey data explained only around 60% of alcohol sales, with the difference between survey and sales data equating to around one bottle of wine per week per adult drinker (aged 16 years and over) [8]. Similar differences have been observed in other countries with the proportions of alcohol sales explained by surveys ranging from, for instance, less than a third of state sales in the USA [12], up to 80% of national sales in Australia [13].

A number of hypotheses have been suggested to explain such differences. These include underestimation of the size of generic drinks reported in surveys, poor recollection, and other reporting biases of consumption by survey participants, and an inability to capture some heavier drinkers in the survey samples [14-16]. To a limited extent, modelled and empirical tests of these hypotheses have been able to explain some of the difference between reported consumption and recorded sales [17,18]. However, surveys often record typical drinking by asking, for instance, consumption on a typical drinking day and then applying this to reported drinking frequency, or by measuring drinking during a recent period (e.g., last week) [9,14]. Such measures may fail to capture atypical consumption with individuals excluding from their responses heavy drinking periods (e.g., Christmas) and periods of abstinence (e.g., Dry January) [19,20]. Moreover, even in surveys running throughout the year, individuals may participate less at certain times (e.g., holiday periods) meaning recent consumption in such periods (e.g., in the last week) can be poorly represented.

Drinking patterns are rarely regular or unchanging, rather, they are characterised by peaks and troughs in consumption depending on weekday, season, and special occasions. Survey methods that attempt to directly measure temporal and contextual changes in individuals’ alcohol consumption provide better measures of alcohol consumption which are more consistent with levels of alcohol sales. Thus, the Graduated-Frequency methodology [21,22] asks individuals how often (e.g., over the past 12 months) they have consumed specified numbers of standard alcohol drinks in one day (e.g., ≥12, 11–8, drinks, etc.). Equally, context-specific methodologies first identify the locations in which an individual consumes alcohol (e.g., home, bars, restaurants), then measure levels of alcohol consumption within each context [18]. Other methods attempt to capture a mix of both typical and atypical consumption by measuring drinking on, for instance, the day before survey (Yesterday method, [13,23]). Inevitably, such methodologies are limited by definitional issues (e.g., how well people understand the concept of a standard drink in Graduated-Frequency), questionnaire length (e.g., to capture all drinking contexts), and the smaller number of drinking occasions captured if a Yesterday type methodology is used alone.

Herein, we describe a new method to measure both typical alcohol consumption and changes in levels of consumption associated with holidays, festivals, sports events, weddings, bereavements, and other special events over a 12-month period (Box 1). We utilise this methodology to undertake a large national survey in a country (England) with a drinking culture historically characterised by low levels of routine drinking interspersed with periods of heavier, social consumption [24-26]. We use results from this survey to correct published national estimates of alcohol consumption in lower-, increasing-, and higher-risk drinkers [9]. Finally, we examine how revised estimates may alter risks of alcohol-related morbidity and mortality in different demographic and consumer groups.

## Methods

### Survey tool

Respondents were asked if they had ever consumed alcohol and if they had drank in the last 12 months. For those responding yes to both questions (classed as current drinkers), detailed information about alcohol consumption was collected in three domains: typical drinking, routine atypical drinking, and special occasion drinking.

#### Typical drinking

Respondents were asked “*On average, how often have you drank alcohol in the last 12 months*”. Responses were recorded as number of days per week or, for those drinking less than weekly, categories of 2 to 3 days per month, 1 day per month, or less often (for analysis this was set to six occasions/year). Participants were then asked a series of questions to describe how much they would normally drink on a typical day when drinking, with the number of drink types and sizes recorded. Consumption was recorded separately for settings inside (private space) and outside (public space) of the home. Mean quantity of alcohol consumed per week (in grams and UK units, with 1 unit approximated to 8 g of pure alcohol) was calculated using the conversion criteria outlined in Box 2. Individuals were classified by sex to categories of typically weekly consumption based on those used by national statistics (HSE; Lower risk, 3 categories; Increasing risk, 2 categories; and Higher risk, 1 category; See Box 3 for definitions [9]).

#### Feasting and fasting

Individuals were first asked if there were days in the last 12 months when they drank a bit more, a lot more, or less than usual. Respondents who acknowledged having such days were asked to estimate how much they drank on these days (using the same methods and conversions; Box 2).

To capture routine atypical days (e.g., heavier drinking every Friday) the frequency of such days was reported (either weekly, monthly, or yearly). In each scenario, individuals also identified if the drinking days were in addition to, or instead of, typical drinking days. Combined with typical drinking days these measures provided a total number of drinking days in a year across the four possible consumption levels (typical, a bit more, a lot more, less) for each respondent (Additional file 1: Table S1).

A number of studies have identified the importance of context-specific questions in order to generate more inclusive measures of alcohol consumption [18,27]. Consequently, a second set of questions asked specifically about special drinking periods (periods of multiple days or weeks, e.g., while on holiday; Box 1) and events (e.g., a wedding) in the last 12 months. For special periods, respondents were asked to identify if they changed their frequency of drinking or the quantity consumed. If either changed, respondents identified how long the period of change lasted, their drinking frequency over the period, and the quantity of alcohol consumed when drinking, based on their previously quantified levels but also including an abstinence option (i.e., abstained, a bit less, a bit more, a lot more). The period identified replaced the same length period in individuals’ routine drinking patterns and annual consumption was recalculated (Additional file 1: Table S1). Finally, for special events (Box 1), respondents indicated any such event in the last 12 months that changed their consumption level, how consumption changed (i.e., abstained, a bit less, a bit more, a lot more), and the number of days on which each type of event had occurred. Although a conservative estimate, the number of special event days was substituted for average consumption days. New annual consumption was calculated (in g/year) and converted into a weekly consumption rate (units/week; Additional file 1: Table S1). This final figure is referred to as the adjusted consumption level.

Additional data collected and used in analyses present here included single year of age (categorised into seven age categories for analysis; Table 1), sex, and ethnicity (collected according to Office for National Statistics groupings [28] and then categorised into White, Asian/Chinese, and Black/Other for the purposes of analysis). Other fields included in the survey measured historical drinking patterns and both current and historical health status, although these are not used in the analyses presented here. The survey was piloted on 840 individuals between November 2012 and February 2013 and minor changes were made to the wording of questions and prompts provided by surveyors in order to improve clarity for potential respondents.

**Table 1:** **Demographics, lifetime abstinence, and frequency of current drinking** ^**a**^ **in the study sample**

		**Total sample**		**Ever drank %**		
				**No**	**Yes**	
					**Last 12 months** ^**a**^	
					**No**	**Yes**
		**n**	**%**	**%**	**%**	**%**
**All**		6,085	100.0	5.9	18.5	75.7
**Sex**	Male	2,160	35.5	4.8	15.3	79.9
	Female	3,925	64.5	6.5	20.2	73.4
				*χ* ^2^ = 32.084, *P* <0.001		
**Age**	16–24	310	5.1	12.3	8.1	79.7
	25–34	463	7.6	7.8	10.4	81.9
	35–44	668	11.0	6.9	17.1	76.0
	45–54	1,043	17.1	4.9	14.7	80.4
	55–64	1,298	21.3	3.6	17.5	78.9
	65–74	1,370	22.5	3.8	22.0	74.2
	75+	933	15.3	9.3	27.3	63.3
				*χ* ^2^ = 187.917, *P* <0.001		
**Deprivation tertile**	Deprived	1,995	32.8	8.6	22.4	69.0
	Mid	1,284	21.1	5.2	19.0	75.8
	Affluent	2,806	46.1	4.2	15.4	80.4
				*χ* ^2^ = 90.886, *P* <0.001		
**Ethnicity** ^**b**^	White	5,660	93.0	3.8	18.3	78.0
	Asian/Chinese	191	3.1	56.0	17.8	26.2
	Black/Other	234	3.8	16.2	23.1	60.7
				*χ* ^2^ = 980.727, *P* <0.001		

^a^Individuals who consumed alcohol in the last 12 months are considered current drinkers; ^b^Black/Other includes those who preferred not to give an ethnicity. Further details of ethnic categories are given in the methods.

#### Sampling

Inclusion criteria were resident in England and aged 16 years or over. Based on national prevalence of typical consumption categories, a target sample size of 6,000 individuals was set in order to achieve around 300 in the lowest prevalence consumption category (i.e., higher risk consumption, 5% males, 4% females, 2012, HSE [9]). The sample used a random probability method delivered through telephone interview (May 2013 to April 2014), using a whole year in order to ensure any calendar effects of consumption were minimised [20]. English landline numbers were randomly selected (by a commercial provider) from a stratified database to provide equal coverage across all English geographical regions. Stratification was not possible for mobile phone numbers. For participant convenience, mobiles were texted prior to being phoned and respondents could opt out of being called at that stage (opt outs from texts <100). Random Digit Dialling [29] was used to call phone numbers. For both landlines and mobiles, surveys recorded respondents’ postcodes and these were converted into lower super output areas (LSOAs; geographical areas with a population mean of 1,500 [30]). LSOAs were then used to allocate each respondent a measure of deprivation using the 2010 Index of Multiple Deprivation – a composite measure including 38 indicators relating to economic, social, and housing issues available for all LSOAs [31]; for the purposes of analysis, the Index of Multiple Deprivation was then categorised into three tertiles of deprivation (Deprived, Mid, Affluent; Table 1).

Phone numbers were called a maximum of seven times (Monday, Wednesday, Friday, 9.30 am to 5.30 pm; Tuesday, Thursday, 9.30 am to 9.00 pm; Saturday, 10.00 am to 4.00 pm). Calls resulting in contacts with fax machines, business addresses, and dead lines were removed. No answers, call back requests, and answer machines were called until a respondent provided either a yes (n = 6,092) or no (n = 20,092), or the study end date was reached. Of the 6,092 completing the survey, 6,085 individuals provided all data items required. Consequently, the response rate was calculated as 23.3% (n = 6,085/26,184).

#### Analysis

Responses were recorded using a computer-assisted telephone interview system with data transferred to SPSS v20 for coding, cleaning, and analysis. χ^2^ and ANOVA were used to examine differences in abstinence and consumption between demographic categories. Generalized Linear Modelling (GLM) was used to measure independent relationships between demographics and changes in consumption identified through enhanced survey measures (i.e., atypical and special consumption), and the best fit model used to generate correction factors from reported typical to adjusted consumption by age, sex, and typical weekly drinking category. Finally, modelled corrections were applied to the England population using age, sex, and nationally reported consumption categories (population [32]; nationally surveyed typical consumption by age and sex [9]). Ethical approval for the study was obtained from Liverpool John Moores University’s Research Ethics Committee.

## Results

Demographics and alcohol drinking behaviours are shown in Table 1. Individuals aged 16 to 24 years were most likely to be lifetime abstainers. Abstinence was also more common in the most deprived individuals and those of Asian/Chinese ethnicity. Being a current (last 12 months) drinker was associated with affluence and being male, white, and under 75 years of age. Further analyses are limited to current drinkers (n = 4,604) and focus on typical consumption (unadjusted units/week), adjusted consumption, and adjustment size (adjusted minus typical consumption).

Amongst drinkers, neither ethnicity nor deprivation were significantly associated with typical weekly consumption, adjusted consumption, or adjustment size (Table 2). Typical weekly and adjusted consumption was higher in males. Males also incurred a larger absolute adjustment (units/week) than females but much smaller adjustment relative to typical consumption (percentage increase, females 29.5%, males 18.3%). Age was also associated with variations in typical weekly consumption, adjusted consumption, and adjustment size with absolute (units) and relative adjustments (percentage change) being highest in those aged 16 to 24 years. After adjustment, those aged 45 to 54 years and 16 to 34 years showed the highest weekly consumption (Table 2). Highest absolute adjustments were not in the highest drinking category but in the second highest group with atypical and special occasions adding 4.2 units (33.8 g) of alcohol consumption per week. However, the largest relative increase was in the lowest drinking category where adjustment nearly tripled estimated weekly consumption (from 0.4 to 1.1 units/week; Table 2). Adjustment resulted in the proportion of respondents in the highest drinking category increasing from 3.6% (Table 2) to 5.3%.

**Table 2:** **Mean alcohol consumed through typical, atypical/special, and all drinking occasions by demographics and typical consumption**

					**UK Units** ^**a**^ **per week**									
					**Typical**			**Atypical/special**			**All**			**% change**
					**95% CI**			**95% CI**			**95% CI**			
			**n**	**%**	**Mean**	**Lower**	**Upper**	**Mean**	**Lower**	**Upper**	**Mean**	**Lower**	**Upper**	
**Total**			4,604		10.0	9.5	10.5	2.3	2.1	2.5	12.3	11.8	12.9	23.0
**Sex**	Male		1,725	37.5	15.6	14.4	16.7	2.8	2.4	3.3	18.4	17.2	19.6	18.3
	Female		2,879	62.5	6.7	6.3	7.1	2.0	1.8	2.2	8.7	8.2	9.2	29.5
	F, *P*				F = 287.303, *P* <0.001			F = 15.990, *P* <0.001			F = 280.921, *P* <0.001			
**Age**	16–24		247	5.4	9.3	7.3	11.3	4.9	3.5	6.2	14.2	11.9	16.5	52.3
	25–34		379	8.2	10.1	7.6	12.7	4.0	3.0	5.0	14.1	11.1	17.1	39.3
	35–44		508	11.0	8.7	7.7	9.7	3.1	2.5	3.8	11.8	10.5	13.2	36.0
	45–54		839	18.2	11.3	10.1	12.5	3.1	2.5	3.6	14.3	12.9	15.8	27.1
	55–64		1,024	22.2	11.2	10.0	12.4	2.2	1.7	2.6	13.4	12.1	14.7	19.3
	65–74		1,016	22.1	9.9	8.9	10.9	1.2	0.8	1.5	11.1	10.1	12.1	11.7
	75+		591	12.8	7.8	6.7	8.9	0.6	0.3	0.8	8.4	7.2	9.5	7.6
	F, P				F = 3.663, *P* = 0.001			F = 22.220, *P* <0.001			F = 7.690, *P* <0.001			
**Deprivation**	Deprived		1,376	29.9	10.2	9.1	11.3	2.1	1.7	2.4	12.2	11.0	13.5	20.2
**tertile**	Mid		973	21.1	10.6	9.5	11.8	2.5	2.0	3.1	13.2	11.9	14.4	23.9
	Affluent		2,255	49.0	9.7	9.1	10.3	2.4	2.1	2.6	12.1	11.4	12.7	24.3
	F, *P*				F = 0.990, *P* = 0.372			F = 1.414, *P* = 0.243			F = 1.122, *P* = 0.326			
**Ethnicity**	White		4,412	95.8	10.1	9.6	10.6	2.3	2.1	2.5	12.4	11.9	13.0	22.9
	Asian/Chinese		50	1.1	5.8	2.6	9.0	1.4	0.4	2.5	7.2	3.9	10.6	25.1
	Black/Other		142	3.1	8.7	6.5	11.0	2.3	1.3	3.4	11.1	8.4	13.8	26.9
	F, *P*				F = 1.876, *P* = 0.153			F = 0.364, *P* = 0.695			F = 2.033, *P* = 0.131			
**Typical alcohol consumption category** ^**b**^	Lower risk	1	1,300	28.2	0.4	0.4	0.4	0.8	0.6	1.0	1.1	0.9	1.3	209.3
		2	1,636	35.5	3.8	3.7	3.9	2.4	2.2	2.7	6.2	5.9	6.5	64.9
		3	820	17.8	12.1	11.8	12.3	3.3	2.8	3.7	15.3	14.8	15.9	27.1
	Increasing risk	4	436	9.5	21.7	21.1	22.3	3.6	2.9	4.3	25.3	24.4	26.3	16.7
		5	247	5.4	32.2	31.1	33.2	4.2	2.8	5.6	36.4	34.6	38.2	13.1
	Higher risk	6	165	3.6	74.2	67.3	81.1	1.9	−1.2	5.1	76.1	68.3	83.9	2.6
	F, *P*				n/a			F = 22.337, *P* < 0.001			F = 1592.674, *P* < 0.001			

^a^1 UK Unit = 8 g of alcohol; ^b^Consumption categories are taken from Health Survey for England [9]; see Box 1. Statistics use Analysis of Variance (ANOVA). 95% CI, 95% Confidence intervals.

To account for confounding between demographics, GLM was employed to model independent levels of adjustment associated with each independent variable. Dependent variables were limited to those showing a significant association with levels of adjustment (Table 2) and all significant main and two-way interactions were included in the model (Table 3). In GLM, age, typical consumption category, and sex were all significantly associated with adjustment level as well as the interactive term between age and consumption category (Table 3). The best-fit model was used to generate adjustment values (units/week) by age, sex, and drinking category (Additional file 2: Table S2). The largest adjustments (additional units/week) were in those aged 25 to 34 years in the heaviest typical drinking category, where adjustment added around 18 additional units/week (144 g/week) to reported typical drinking for both males and females. Those aged 16 to 24 years in the third highest drinking category and those aged 35 to 44 years in the second highest also showed substantial positive adjustments (Additional file 2: Table S2). Not all adjustments were positive with, for instance, consumption falling by 4.6 units/week in females aged 65 to 74 years in the highest drinking category once atypical/special consumption had been taken into account. Most negative adjustments were seen in the heaviest drinkers aged 55 years or over (Additional file 2: Table S2).

**Table 3:** **Generalized Linear Model of additional units consumed per week through atypical and special occasion drinking**

				**95% CIs**			
**Variable**		**Category**	**B**	**Lower**	**Upper**	***χ*** ^**2**^	***P***
**Sex**		Male	0.595	0.176	1.014	7.746	0.005
		Female	Ref				
**Age**		16–24	−1.035	−6.480	4.410	0.139	0.709
		25–34	19.322	14.108	24.536	52.750	<0.001
		35–44	3.649	−1.570	8.868	1.877	0.171
		45–54	10.815	6.497	15.132	24.101	<0.001
		55–64	0.015	−4.271	4.302	0	0.994
		65–74	−2.807	−7.013	1.400	1.710	0.191
		75+	Ref	(χ^2^ = 200.013, *P* <0.001)			
**Typical alcohol consumption category** ^**a**^	Lower risk	1	1.786	−2.008	5.580	0.851	0.356
		2	2.343	−1.470	6.155	1.450	0.228
		3	2.392	−1.564	6.348	1.405	0.236
	Increasing risk	4	3.589	−0.685	7.862	2.709	0.100
		5	0.962	−3.554	5.479	0.174	0.676
	Higher risk	6	Ref	(χ^2^ = 104.936, *P* <0.001)			
**Interactions** ^**b**^	Age × Consumption			(χ^2^ = 249.211, *P* <0.001)			
	Age × Sex			(χ^2^ = 3.277, *P* = 0.773)			
	Consumption × Sex			(χ^2^ = 7.688, *P* = 0.174)			

^a^Prior to adjustment for atypical/special alcohol consumption; see Box 1. Non-significant terms were removed from the model. Figures in brackets represent overall statistical significance of variable when independent variable has more than two categories. ^b^Only overall statistics are presented for interactions due to space constraints. 95% CI, 95% Confidence intervals; Ref, Reference category.

HSE provides national estimates of non-drinkers and proportions in each typical consumption category by sex and the same age categories used here [9]. These were combined with national population statistics for England to generate total populations by age, sex, and typical consumption category (Additional file 3: Table S3). Table 4 presents adjusted weekly alcohol consumption by age and typical consumption category stratified by sex (adjustment data from Additional file 2: Table S2 weighted by population data from Additional file 3: Table S3). Post-adjustment, males aged 25 to 34 years show the highest weekly consumption. However, males in the lowest alcohol consumption category see the largest relative consumption increase; from 0.4 to 1.8 units/week (Table 4). Amongst women, the age group showing the largest relative increase in consumption was those aged 16 to 24 years, where atypical and special occasion drinking increased consumption by over 50% (from 10.2 to 15.5 units/week). Furthermore, in all but the lowest risk drinking category, relative increases in units per week were higher in women than men (Table 4).

**Table 4:** **Modelled, population-weighted alcohol consumed through typical, atypical/special, and all drinking occasions**

			**England population drinkers**		**UK Units** ^**a**^ **per week**				**Population UK Units per week**	
					**Typical**	**Atypical special**	**Fully adjusted**	**% Change** ^**b**^		
**Category**			**n (1,000s)**	**%**					**Millions**	**%** ^**c**^
Male drinkers by age (years)	16–24		2,550.8	7.1	15.3	6.4	21.6	41.7	55.2	9.0
	25–34		3,157.2	8.8	18.0	5.3	23.3	29.6	73.6	12.0
	35–44		3,139.4	8.7	17.0	3.8	20.8	22.6	65.4	10.6
	45–54		3,245.2	9.0	18.7	4.0	22.8	21.5	73.9	12.0
	55–64		2,657.5	7.4	19.7	2.7	22.3	13.6	59.3	9.6
	65–74		2,683.8	7.4	17.6	1.7	19.3	9.7	51.7	8.4
	75+		1,307.2	3.6	11.4	1.1	12.5	9.7	16.4	2.7
All males	≥16		18,741.1	52.0	17.3	3.8	21.1	22.0	395.4	64.3
Female drinkers by age (years)	16–24		2,343.5	6.5	10.2	5.3	15.5	51.6	36.4	5.9
	25–34		2,921.9	8.1	9.3	4.2	13.5	45.5	39.4	6.4
	35–44		3,041.9	8.4	9.6	3.5	13.1	37.0	39.8	6.5
	45–54		3,162.1	8.8	10.4	3.1	13.5	29.6	42.6	6.9
	55–64		2,500.8	6.9	12.1	1.9	14.1	15.8	35.2	5.7
	65–74		1,944.9	5.4	8.5	1.0	9.5	11.6	18.5	3.0
	75+		1,416.0	3.9	5.4	0.3	5.8	6.3	8.2	1.3
All females	≥16		17,331.1	48.0	9.7	3.0	12.7	31.3	220.0	35.7
Male drinkers by consumption category^d^	Lower risk	1	1,864.0	5.2	0.4	1.4	1.8	315.7	3.3	0.5
		2	7,434.5	20.6	4.4	3.4	7.8	77.8	58.3	9.5
		3	4,250.1	11.8	14.7	4.1	18.8	28.0	79.8	13.0
	Increasing risk	4	2,945.2	8.2	27.6	5.4	33.0	19.5	97.1	15.8
		5	1,110.6	3.1	40.6	5.3	45.8	13.0	50.9	8.3
	Higher risk	6	1,136.6	3.2	89.8	3.6	93.3	4.0	106.1	17.2
Female drinkers by consumption category^d^	Lower risk	1	3,462.5	9.6	0.4	0.7	1.1	193.5	3.7	0.6
		2	6,992.4	19.4	3.2	2.8	6.1	88.4	42.5	6.9
		3	2,978.5	8.3	9.9	3.6	13.5	36.9	40.3	6.5
	Increasing risk	4	1,446.4	4.0	16.7	5.1	21.8	30.3	31.6	5.1
		5	1,538.9	4.3	26.7	5.4	32.1	20.2	49.4	8.0
	Higher risk	6	912.4	2.5	53.8	3.8	57.6	7.1	52.6	8.5
Total all drinkers			36,072.2	100.0	13.6	3.4	17.1	25.1	615.4	100.0

^a^1 UK Unit = 8 g of pure alcohol; ^b^Percentage change is atypical/special consumption as proportion of typical; ^c^Percentage represents the proportion of all units consumed nationally for each demographic category; ^d^See Box 1 for definitions.

The total units of alcohol cleared for sale in England (calculated from total UK sales and proportions of each alcohol type sold in England; 2013 [10]) are equivalent to 783.8 million units per week (excludes 3.6 million units consumed by 11- to 15-year-olds; Table 5). The 2012 HSE [9] accounts for 63.2% of these sales through weighted reported consumption (based on typical drinking) and our measure of typical drinking (uncorrected) accounts for approximately the same (62.7%). Using adjusted measures, however, total units consumed in those ≥16 years was 615.44 million units/week; a mean consumption of 17.1 units/drinker/week (or 136.5 g/week, Table 5). This is equivalent to 78.5% of the 783.8 million units cleared (taxed) for sale in 2013.

**Table 5:** **Weekly alcohol consumption in drinkers** ^**a**^ ≥**16 years – health survey for England, special occasion survey and sales**

			**UK Units** ^**b**^ **/week**		**% of HMRC sales**
		**Population (1,000s)**	**Total (millions)**	**Per drinker**	
**HMRC** ^**c**^ (sales)	All	36,072.2	783.8	21.7	NA
**Health Survey for England**	Male	18,741.1	318.6	17.0	40.6
	Female	17,331.1	176.8	10.2	22.6
	All	36,072.2	495.4	13.7	63.2
**ARUK Survey**	Male	18,741.1	324.2	17.3	41.4
Typical drinking only	Female	17,331.1	167.6	9.7	21.4
	All	36,072.2	491.8	13.6	62.7
**ARUK Survey**	Male	18,741.1	395.4	21.1	50.5
Typical/atypical and special occasion	Female	17,331.1	220.0	12.7	28.1
	All	36,072.2	615.4	17.1	78.5

^a^Number of drinkers are based on Health Survey for England 2012 [9]; ^b^1 UK unit = 8 g of pure alcohol; ^c^ HMRC, Her Majesties Revenue and Customs – clearance data measures total alcohol taxed for sale across the UK (2013 [11]). The British Beer and Pub Association [10] publishes proportions sold regionally allowing the calculation of sales for England & Wales only. Total sales for England were then based on a per capita split between England and Wales (2012 [32]). To provide a comparable figure to survey consumption by ≥16 years only, total sales were further reduced by 190,982,542 units based on estimated annual consumption by those 11 to 15 years (2012 [48]). NA, Not applicable.

## Discussion

Festive drinking, often as a counterpoint to the norms of daily sobriety, has a long history in many societies [33]. Recent marketing techniques have capitalised on established drinking occasions (e.g., New Year) and actively encouraged greater associations between alcohol consumption and major sporting events, established holidays (e.g., Halloween), and practically any personal occasion for celebration [34]. With so many heavily promoted special drinking occasions, measuring individuals’ typical drinking is increasingly likely to omit major elements of their consumption patterns. For England, we have shown that including survey questions on atypical and special occasion consumption increases estimates of average weekly drinking by nearly a quarter (13.6 to 17.1 units/person/week; Table 5). The increase is, however, not equally distributed, with the impact of adjustment generally decreasing with age in both males and females and, for instance, increasing units/week in 16- to 24-year-old females by more than 50% (Table 4). Younger drinkers often have expendable income and opportunity to incorporate weekly binges and increased alcohol consumption with celebrations of frequent special occasions [35]. Thus, for the highest risk drinkers aged 25 to 34, accounting for atypical and special occasion drinking was estimated to increase typical weekly consumption by around 18 units (Additional file 2: Table S2). Overall, by broad categories of typical consumption, increasing risk drinkers (of both sexes) showed the greatest number of additional units; adding more than the equivalent of half a bottle of wine per week to typical consumption. However, even for women in the third lowest drinking category (whose typical drinking averages around the equivalent of a bottle of wine/week; 9.9 units), accounting for atypical and special occasion drinking can add an average of 3.6 units/week (Table 4).

The additional units associated with atypical and special occasion drinking can be disproportionately damaging to health. Thus, atypical and special occasion drinking is often associated with bingeing and, therefore, with increased risks of unintentional injury and violence as well as overdose and alcohol poisoning [36]. Further, while the benefits of alcohol consumption on reducing risk of ischaemic heart disease remain contested, even potential cardio-protective benefits are absent when individuals binge once a month or more (>60 g of pure alcohol in one session [37]). Consequently, the impact of over 120 million units of alcohol per week being consumed through atypical and special occasion drinking can both harm health directly and adversely impact on potential cardio-vascular benefits. However, relationships between levels of alcohol consumption and associated harms may also need re-examining where studies have not accounted for atypical and special occasion drinking.

Even with special occasion drinking taken into account, 4.6 units/drinker/week (around half bottle of wine, 12% alcohol by volume; two pints of beer, 4% alcohol by volume) remain unaccounted for in sales data. Other hypotheses may account for this inconsistency [38], such as the fact that illegal immigration may account for over half a million individuals [39]; although their consumption patterns are unknown. Additionally, individuals institutionalised in health, social, and judicial establishments or homeless are often omitted from alcohol surveys and would not have been captured here. However, while they may be heavier consumers of alcohol, they typically account for a small proportion of the population [40]. Tourists may consume substantial amounts of alcohol – although such consumption is, in part, counterbalanced by English individuals drinking abroad [8,41]. Some alcohol may also be thrown away but this is unlikely to account for over 168 million units/week, even without factoring in units missing from UK sales data (e.g., illegal alcohol imports and home brew) [17]. What remains likely is that individuals continue to underestimate how much they consume, especially through home poured spirits and wine [42], and may also fail to report some drinking occasions either accurately or at all.

### Limitations

Although randomised, the survey did not attempt to generate a representative sample of alcohol consumers and abstainers on a national basis, but instead used national population estimates and stratified drinking survey data to weight responses to the English population. With this survey acting as a proof of concept, a larger nationally representative survey is now required to test the independent utility of this methodology as a national monitoring tool. For special drinking events (Box 1) we were also unable to distinguish if they were instead of or as well as other drinking days. Here, we opted for a conservative measure by removing an average drinking day’s consumption for each special event day reported. How often such occasions should be considered additional consumption requires further study. Participation rates were 23.3% of those contacted and informed about the study and our sample over-represented females, older individuals, and those of white ethnicity (Table 1). The final data were weighted to match national population age and gender demographics (Additional file 3: Table S3). While we did not weight for ethnicity, this demographic was not significantly related to changes in consumption associated with atypical and special occasion drinking. However, even within demographic categories we could not measure whether, for instance, heavier drinkers were less likely to be home or agree to participate in the survey when surveyors called. Our study, like others, was also limited by self-reported drinking and required people recalling drinking over a 12-month period. Although we adapted typical methodologies to more accurately measure self-poured glasses of wine (Box 2), individuals may still have underestimated the size of home poured drinks.

## Conclusions

For centuries, drinking cultures in many nations (including England) have been typified by lower levels of routine alcohol consumption interspersed with heavier social consumption [24,33]. This prevailing culture of heavy drinking on festivals, holidays, and other special occasions, now combined with alcohol promotion that exploits such associations [43], means special occasion drinking is a critical component of alcohol epidemiology. Measuring atypical and special drinking occasions accounts for 41.6% of the gap between national surveyed alcohol consumption and national alcohol sales in England (Table 5). In part, the impact of atypical and special occasion drinking is reflected in evening presentations to emergency units which peak on weekends but also sports events, bank holidays, and even commemorative occasions such as Halloween [44]. Such presentations are more frequent in younger individuals whose alcohol consumption is impacted by frequent parties, weekend binges, and other heavily promoted drinking opportunities (e.g., national football events [44,45]). As well as young drinkers, those drinking above national health guidelines also add considerably to weekly alcohol consumption through atypical and special occasions. An additional 5 units of alcohol per week for males whose typical weekly consumption is 27.6 units (Table 4) may substantially increase lifetime risk of death from alcohol-related disease and injury especially when additional units form parts of binges [46].

Disproportionate underestimation of alcohol consumption by infrequent drinkers has been identified elsewhere [23]. Consistent with such findings, we found that, for very low alcohol consumers, the majority of alcohol is consumed on atypical and special occasions (Table 4). After accounting for such occasions their average consumption typically remained in lower risk drinking categories. However, recent studies have reported infrequent drinkers as having a high probability of presenting at emergency units after those atypical occasions on which they do consume alcohol [47]. Thus, for many individuals, typical drinking alone can be a poor proxy for actual consumption, a poor measure of alcohol-related risks to immediate and long-term health, and consequently of questionable benefit when monitoring consumption trends. Better population measures of celebratory, festival, and holiday drinking are required to measure the full extent of harms caused by alcohol and to ensure that such harms are not discounted or even dismissed by the public.

## Box 1: Measuring changes in alcohol consumption associated with special occasions

Participants were asked if their frequency of alcohol consumption or amount consumed when drinking changed from their reported typical on any of the occasions and events listed below. For special periods, those indicating changes in consumption level and/or frequency were asked how often they drank (e.g., days per week), how much they drank (abstained, bit less, bit more, lot more), and for how long each occasion lasted (e.g., number of weeks). For events, changes in level of consumption associated with the event type were recorded (as special occasions) and then for each event the number of times (days) on which they had occurred in the last 12 months recorded.

Special periods

- Period around Christmas and New Year
- In the summer
- When on holiday at home
- When on holiday away from home
- Bank holiday weekends
- Working away from home
- In January (after New Year)
- During other religious periods, e.g., Lent, Ramadan
- After a bereavement or funeral
- During periods of unemployment or other change in work patterns

Special events

- When celebrating own, a friend’s, or a close relative’s birthday
- At a wedding
- At an engagement, hen, or stag party
- Watching an important sporting event on TV or live
- When friends came to stay or you stayed with friends
- At a festival, rock or pop concert, or other show

## Box 2: Alcohol content conversions with example drink sizes

Beer and Cider ^a^

- Low strength, 2.8% ABV, e.g., Regular bottle, 330 mL, 0.9 UK units, 7.4 g
- Medium strength, 4.5% ABV, e.g., Regular can, 440 mL, 2.0 UK units, 15.8 g
- High strength, 6.5% ABV, e.g., Pint, 568.3 mL, 3.7 UK units, 29.6 g

Wine ^b^

- 12.5% ABV, e.g., Medium glass, 175 mL, 2.2 UK units, 17.5 g

Fortified Wine/Liqueur ^c^

- 17.0% ABV, e.g., Double, 50 mL, 0.9 UK units, 6.8 g

Spirits ^c^

- 40.0% ABV, e.g., Single, 25 mL, 1.0 UK units, 8.0 g

Alcopops ^d^

- 4.0% ABV, e.g., Small bottle, 275 mL, 1.1 UK units, 8.8 g

Cocktails

- 40.0%, One size, 50 mL, 2.0 UK units, 16.0 g

^a^ For beer and cider, small and regular bottle and can size were recorded as well as litre and half litre options. ^b^ As well as three glass sizes (small 125 mL, medium 175 mL, and large 250 mL), home drinking respondents were asked how many glasses they normally get from a standard bottle (750 mL) in order to calibrate their home poured glass size. Respondents could also report consumption of wine in fractions of a bottle (e.g., 1/3 of a 700 mL bottle of wine per day). ^c^ For spirits, liqueurs, and fortified wines, individuals identifying drink sizes above doubles were set as triples. Individuals could also report consumption as a fraction of a bottle size. ^d^ For alcopops, both small (275 mL) and large (700 mL) bottles were recorded. ABV, Alcohol by volume; g, grams of pure alcohol.

## Box 3: Typical weekly drinking category definitions

1. (Lower Risk)
  Males, ≤1 UK unit/week, ≤8 g/week
  Females, ≤1 UK unit/week, ≤8 g/week
2. (Lower Risk)
  Males, >1–10 UK units/week, >8–80 g/week
  Females, >1–7 UK units/week, >8–56 g/week
3. (Lower Risk)
  Males, >10–21 UK units/week, >80–168 g/week
  Females, >7–14 UK units/week, >56–112 g/week
4. (Increasing Risk)
  Males, >21–35 UK units/week, >168–280 g/week
  Females, >14–21 UK units/week, >112–168 g/week
5. (Increasing Risk)
  Males, >35–50 UK units/week, >280–400 g/week
  Females, >21–35 UK units/week, >168–280 g/week
6. (Higher Risk)
  Males, >50 UK units/week, >400 g/week
  Females, >35 UK units/week, >280 g/week

Typical weekly drinking categories based on Health Survey for England [9].

## Additional files

Additional file 1: Table S1. Worked example of corrections to unadjusted typical consumption to include atypical/special occasions. Additional file 2: Table S2. Generalised Linear Model estimates for additional units of alcohol associated with atypical/special occasion drinking [9]. Additional file 3: Table S3. England population stratified by age, sex, and typical weekly consumption category [9,32].

## Abbreviations

GLM: Generalized Linear Modelling
HSE: Health Survey for England
LSOAs: Lower super output areas

## References

[1] World Health Organization (2014). Global status report on alcohol and health 2014.

[2] Rehm J, Mathers C, Popova S, Thavorncharoensap M, Teerawattananon Y, Patra J (2009). Global burden of disease and injury and economic cost attributable to alcohol use and alcohol-use disorders. Lancet..

[3] Rehm J, Shield KD (2014). Global alcohol-attributable deaths from cancer, liver cirrhosis, and injury in 2010. Alcohol Res..

[4] Corrao G, Bagnardi V, Zambon A, La Vecchia C (2004). A meta-analysis of alcohol consumption and the risk of 15 diseases. Prev Med..

[5] Baliunas DO, Taylor BJ, Irving H, Roerecke M, Patra J, Mohapatra S (2009). Alcohol as a risk factor for type 2 diabetes: a systematic review and meta-analysis. Diabetes Care..

[6] Patra J, Taylor B, Irving H, Roerecke M, Baliunas D, Mohapatra S (2010). Alcohol consumption and the risk of morbidity and mortality for different stroke types - a systematic review and meta-analysis. BMC Public Health..

[7] Roerecke M, Rehm J (2012). The cardioprotective association of average alcohol consumption and ischaemic heart disease: a systematic review and meta-analysis. Addiction..

[8] Bellis MA, Hughes K, Cook PA, Morleo M (2009). Off measure: how we underestimate the amount we drink.

[9] Health & Social Care Information Centre. Health Survey for England 2012: health, social care and lifestyles. Leeds: Health and Social Care Information Centre; 2013.

[10] Tettenborn M (2014). British Beer and Pub Association statistical handbook 2014.

[11] HM Revenue & Customs. Alcohol factsheet 2012–13. London: HM Revenue & Customs; 2013.

[12] Nelson DE, Naimi TS, Brewer RD, Roeber J (2010). US state alcohol sales compared to survey data, 1993–2006. Addiction..

[13] Stockwell T, Zhao J, Chikritzhs T, Greenfield T (2008). What did you drink yesterday? Public health relevance of a recent recall method used in the 2004 Australian National Drug Strategy Household Survey. Addiction..

[14] Gmel G, Rehm J (2004). Measuring alcohol consumption. Contemporary Drug Problems..

[15] Livingston M, Callinan S (2015). Underreporting in alcohol surveys: whose drinking is underestimated?. J Stud Alcohol Drugs..

[16] Boniface S, Kneale J, Shelton N (2013). Actual and perceived units of alcohol in a self-defined “usual glass” of alcohol drinks in England. Alcohol Clin Exp Res..

[17] Meier PS, Meng Y, Holmes J, Baumberg B, Purshouse R, Hill-McManus D (2013). Adjusting for unrecorded consumption in survey and per capita sales data: quantification of impact on gender- and age-specific alcohol-attributable fractions for oral and pharyngeal cancers in Great Britain. Alcohol Alcohol..

[18] Casswell S, Huckle T, Pledger M (2002). Survey data need not underestimate alcohol consumption. Alcohol Clin Exp Res..

[19] Kushnir V, Cunningham JA (2014). Event-specific drinking in the general population. J Stud Alcohol Drugs..

[20] Lemmens PH, Knibbe RA (1993). Seasonal variation in survey and sales estimates of alcohol consumption. J Stud Alcohol..

[21] Greenfield TK, Kerr WC, Bond J, Ye Y, Stockwell T (2009). Graduated frequencies alcohol measures for monitoring consumption patterns: results from an Australian national survey and a US diary validity study. Contemporary Drug Problems..

[22] World Health Organization Department of Mental Health and Substance Dependence (2000). International guide for monitoring alcohol consumption and related harm.

[23] Stockwell T, Zhao J, Macdonald S (2014). Who under-reports their alcohol consumption in telephone surveys and by how much? An application of the ‘yesterday method’ in a national Canadian substance use survey. Addiction..

[24] Nicholls J (2009). The politics of alcohol: a history of the drink question.

[25] Room R. Responses in alcohol-related problems in international perspective: characterizing and explaining cultural wetness and dryness. In: La ricerca Italiana sulle bevande alcoliche nel confronto internazionale. Italy: Santo Stefano Belbo (CN); 1989.

[26] Plant M, Plant M (2006). Binge Britain: alcohol and the national response.

[27] Single E, Wortley S (1994). A comparison of alternative measures of alcohol consumption in the Canadian National Survey of alcohol and drug use. Addiction..

[28] Office for National Statistics (2011). Population estimates by ethnic group 2002–2009.

[29] Callegaro M, Ayhan O, Gabler S, Haeder S, Villar A (2011). Combining landline and mobile phone samples: a dual frame approach.

[30] Bates A (2006). Methodology used for producing ONS’s small area population estimates. Popul Trends..

[31] Department for Communities and Local Government (2011). English indices of deprivation 2010.

[32] Office for National Statistics. Population estimates for UK, England and Wales, Scotland and Northern Ireland, Mid 2012. http://www.ons.gov.uk/ons/publications/re-reference-tables.html?edition=tcm%3A77-319259. 2013.

[33] McGovern P (2011). Uncorking the past: the quest for wine, beer and other alcoholic beverages.

[34] Nicholls J (2012). Everyday, everywhere: alcohol marketing and social media – current trends. Alcohol Alcohol..

[35] Seaman P, Ikegwuonu T (2010). Drinking to belong: understanding young adults’ alcohol use within social networks.

[36] Taylor B, Irving H, Kanteres F, Room R, Borges G, Cherpitel C (2010). The more you drink the harder you fall: a systematic review and meta-analysis of how acute alcohol consumption and injury or collision risk increase together. Drug Alcohol Depend..

[37] Roerecke M, Rehm J (2010). Irregular heavy drinking occasions and risk of ischemic heart disease: a systematic review and meta-analysis. Am J Epidemiol..

[38] Boniface S, Shelton N (2013). How is alcohol consumption affected if we account for under-reporting? A hypothetical scenario. Eur J Pub Health..

[39] Gordon I, Scanlon K, Travers T, Whitehead C (2009). Economic impact on the London and UK economy of an earned regularisation of irregular migrants to the UK.

[40] Shield KD, Rehm J (2012). Difficulties with telephone-based surveys on alcohol in high-income countries: the Canadian example. Int J Methods Psychiatr Res..

[41] Hughes K, Bellis MA, Calafat A, Blay N, Kokkevi A, Boyiadji G (2011). Substance use, violence, and unintentional injury in young holidaymakers visiting Mediterranean destinations. J Travel Med..

[42] Kerr WC, Greenfield TK, Tujague J, Brown SE (2005). A drink is a drink? Variation in the amount of alcohol contained in beer, wine and spirits drinks in a US methodological sample. Alcohol Clin Exp Res..

[43] Hastings G, Brooks O, Stead M, Angus K, Anker T, Farrell T (2010). Failure of self-regulation of UK alcohol advertising. BMJ..

[44] Bellis MA, Jarman I, Hughes K, Wyke S, Quigg Z, Luke C (2012). Nighttime assaults: using a national emergency department monitoring system to predict occurrence, target prevention and plan services. BMC Public Health..

[45] Quigg Z, Hughes K, Bellis MA (2013). Effects of the 2010 World Cup football tournament on emergency department assault attendances in England. Eur J Pub Health..

[46] Rehm J, Room R, Taylor B (2008). Method for moderation: measuring lifetime risk of alcohol-attributable mortality as a basis for drinking guidelines. Int J Methods Psychiatr Res..

[47] Ye Y, Bond J, Cherpitel C, Stockwell T, Macdonald S, Rehm J (2013). Risk of injury due to alcohol – evaluating potential bias using the case-crossover usual-frequency method. Epidemiology..

[48] Fuller E, Henderson H, Nass L, Payne C, Phelps A, Ryley A (2013). Smoking, drinking and drug use among young people in England in 2012.

