# Supplementary material for: Holidays, celebrations, and commiserations: measuring drinking during feasting and fasting to improve national and individual estimates of alcohol consumption
Source: BMC Med. 2015 May 22;13:113. doi: 10.1186/s12916-015-0337-0 (PMC4494693; doi:10.1186/s12916-015-0337-0)
Supplement: Additional file 1: Table S1. — Worked example of corrections to unadjusted typical consumption to include atypical/special occasions. [file 12916_2015_337_MOESM1_ESM.docx]

**Supplementary table 1: Worked example of corrections to unadjusted typical consumption to include atypical/special occasions**

|  | Typical and relative drinking levels^i^ | | | | | Days | | Consumption | |
| --- | --- | --- | --- | --- | --- | --- | --- | --- | --- |
|  | Typical | Bit more | Lot more | Bit less | Abstinent | Drinking | Total | Gms/year | Units/week |
| Reported Consumption (gms/drinking day) | 20 | 30 | 60 | 8 | 0 |  |  |  |  |
| **Unadjusted Typical** |  |  |  |  |  |  |  |  |  |
| a) Drinking frequency (days/year) | 156 | na | na | na | 209 | 156 | 365 | 3129 | 7.5 |
| Adjustment for routine atypical days |  |  |  |  |  |  |  |  |  |
| Reported as replacing typical days | na | yes | no | yes | na |  |  |  |  |
| b) Adjusted drinking frequency (days/year)^ii^ | 52 | 52 | 12 | 52 | 197 | 168 | 365 | 3744 | 9.0 |
| **Adjustment for special drinking periods^iii^** |  |  |  |  |  |  |  |  |  |
| When on holiday away from home |  | 20 |  |  | 8 | 20 | 28 | 600 |  |
| January after New Year |  |  |  |  | 31 | 0 | 31 | 0 |  |
| All special periods resulting in changed drinking |  | 20 |  |  | 39 | 20 | 59 | 600 |  |
| Adjusted typical, excl. special periods^iv^ | 44 | 44 | 10 | 44 | 165 | 141 | 306 | 3139 |  |
| c) Adjusted typical, incl. special periods^i^ | 44 | 64 | 10 | 44 | 204 | 161 | 365 | 3739 | 9.0 |
| **Adjustment for special drinking events** |  |  |  |  |  |  |  |  |  |
| Celebrating own, friend’s or close relative's birthday |  |  | 8 |  |  | 8 | 8 | 480 |  |
| Watching special sporting occasion on TV or live |  | 3 |  |  |  | 3 | 3 | 90 |  |
| All special drinking occasions resulting in changed drinking |  | 3 | 8 |  |  | 11 | 11 | 570 |  |
| Adjusted for special drinking periods excl. special events^v^ | 41 | 59 | 9 | 41 | 204 | 150 | 354 | 3484 |  |
| d) Adjusted for special drinking periods excl. special events^v^ | 41 | 62 | 17 | 41 | 204 | 161 | 365 | 4054 | 9.7 |

i Relative drinking levels are reported by individuals by comparison to typical consumption with respondents detailing the size and types of drinks consumed in each category (see Box 1). ii Frequency of typical drinking is corrected by removing all days drinking at other levels reported as instead of typical drinking days. iii When on holiday away from home and January after New Year were identified as examples of increased and decreased special drinking periods respectively. See Box 1 for full list. iv Adjusted typical drinking days are reduced by subtracting the periods of the year identified as special drinking periods. Days drinking are calculated from the frequency of drinking and the relative drinking level reported for that period. v Total number of drinking days are reduced by number reported for special drinking events and number at each drinking level rebased according to distribution in row c). For more details see methods. All figures are rounded to nearest whole number except for units/week.
