# Supplementary material for: Holidays, celebrations, and commiserations: measuring drinking during feasting and fasting to improve national and individual estimates of alcohol consumption
Source: BMC Med. 2015 May 22;13:113. doi: 10.1186/s12916-015-0337-0 (PMC4494693; doi:10.1186/s12916-015-0337-0)
Supplement: Additional file 2: Table S2. — Generalised Linear Model estimates for additional units of alcohol associated with atypical/special occasion drinking [9]. [file 12916_2015_337_MOESM2_ESM.docx]

**Supplementary table 2: Generalised Linear Model estimates for additional units of alcohol associated with atypical/special occasion drinking**

|  |  | | | | **Age group** | | | | | | | | | | | | | |
| --- | --- | --- | --- | --- | --- | --- | --- | --- | --- | --- | --- | --- | --- | --- | --- | --- | --- | --- |
|  | **Typical weekly drinking category** | | | | **16-24** | | **25-34** | | **35-44** | | **45-54** | | **55-64** | | **65-74** | | **75+** | |
|  | **Consumption*** | | **Units**# | **Grams** | **Mean** | **SE** | **Mean** | **SE** | **Mean** | **SE** | **Mean** | **SE** | **Mean** | **SE** | **Mean** | **SE** | **Mean** | **SE** |
| **Male** | Lower risk | 1 | 1 or less | >=8 | 2.7 | 0.9 | 1.5 | 0.7 | 1.2 | 0.6 | 1.4 | 0.5 | 1.6 | 0.5 | 0.9 | 0.4 | 0.6 | 0.5 |
|  |  | 2 | >1 to 10 | >8-80 | 6.1 | 0.6 | 4.2 | 0.6 | 3.4 | 0.5 | 2.5 | 0.4 | 2.4 | 0.4 | 2.2 | 0.4 | 1.2 | 0.5 |
|  |  | 3 | >10 to 21 | >80-168 | 6.5 | 1.1 | 4.9 | 0.8 | 4.2 | 0.7 | 4.7 | 0.5 | 3.6 | 0.5 | 2.2 | 0.5 | 1.2 | 0.7 |
|  | Increasing risk | 4 | >21-35 | >168-280 | 13.9 | 2.0 | 7.5 | 1.4 | 3.5 | 0.9 | 4.3 | 0.7 | 3.7 | 0.6 | 2.5 | 0.8 | 2.4 | 1.1 |
|  |  | 5 | >35-50 | >280-400 | 3.4 | 1.9 | 4.8 | 1.8 | 15.2 | 1.4 | 6.4 | 0.9 | 3.8 | 0.8 | 1.6 | 1.0 | -0.2 | 1.3 |
|  | Higher risk | 6 | >50 | >400 | -2.2 | 2.0 | 18.2 | 1.9 | 2.5 | 1.9 | 9.7 | 1.1 | -1.1 | 1.1 | -4.0 | 1.0 | -1.2 | 1.9 |
| **Female** | Lower risk | 1 | 1 or less | >=8 | 2.1 | 0.9 | 0.9 | 0.7 | 0.6 | 0.6 | 0.8 | 0.5 | 1.0 | 0.4 | 0.3 | 0.4 | 0.0 | 0.4 |
|  |  | 2 | >1-7 | >8-56 | 5.5 | 0.6 | 3.6 | 0.6 | 2.8 | 0.5 | 2.0 | 0.4 | 1.8 | 0.4 | 1.6 | 0.4 | 0.6 | 0.5 |
|  |  | 3 | >7-14 | >56-112 | 5.9 | 1.1 | 4.3 | 0.8 | 3.6 | 0.7 | 4.1 | 0.5 | 3.0 | 0.5 | 1.6 | 0.5 | 0.6 | 0.7 |
|  | Increasing risk | 4 | >14-21 | >112-168 | 13.3 | 2.1 | 6.9 | 1.4 | 2.9 | 0.9 | 3.7 | 0.7 | 3.1 | 0.6 | 1.9 | 0.8 | 1.8 | 1.1 |
|  |  | 5 | >21-35 | >168-280 | 2.8 | 1.9 | 4.2 | 1.8 | 14.6 | 1.4 | 5.8 | 0.9 | 3.2 | 0.8 | 1.0 | 1.0 | -0.8 | 1.3 |
|  | Higher risk | 6 | >35 | >280 | -2.8 | 2.1 | 17.6 | 1.9 | 1.9 | 1.9 | 9.1 | 1.2 | -1.7 | 1.1 | -4.6 | 1.0 | -1.8 | 1.9 |

*Typical weekly alcohol consumption (prior to adjustment for atypical and special consumption); see Box 1. Consumption categories are based on those published in the Health Survey for England.[9] #Units are UK units of alcohol consumption and are equivalent to 8 grams of alcohol. Model included the independent variables age, sex and consumption category (see Methods for more details). SE=Standard Error.
