# Supplementary material for: Holidays, celebrations, and commiserations: measuring drinking during feasting and fasting to improve national and individual estimates of alcohol consumption
Source: BMC Med. 2015 May 22;13:113. doi: 10.1186/s12916-015-0337-0 (PMC4494693; doi:10.1186/s12916-015-0337-0)
Supplement: Additional file 3: Table S3. — England population stratified by age, sex, and typical weekly consumption category [9,32]. [file 12916_2015_337_MOESM3_ESM.docx]

**Supplementary table 3: England population stratified by age, sex and typical weekly consumption category**

|  |  | |  | |  | | **Age** | | | | | | | | | | | | | | | | | | | | |
| --- | --- | --- | --- | --- | --- | --- | --- | --- | --- | --- | --- | --- | --- | --- | --- | --- | --- | --- | --- | --- | --- | --- | --- | --- | --- | --- | --- |
|  | **Weekly drinking category** | | | | | | **16-24** | | | **25-34** | | | **35-44** | | | **45-54** | | | **55-64** | | | **65-74** | | | **75+** | | |
|  | **Category*** | | | **Units** | | **Grams** | **%** | **n (1000s)** | | **%** | **n (1000s)** | | **%** | **n (1000s)** | | **%** | **n (1000s)** | | **%** | **n (1000s)** | | **%** | **n (1000s)** | | **%** | **n (1000s)** | |
| **Male** | Abstainer |  | | 0 | | 0 | 20 | | 637.7 | 13 | | 466.4 | 13 | | 469.1 | 12 | | 442.5 | 11 | | 328.5 | 13 | | 401.0 | 20 | | 326.8 |
|  | Lower risk | 1 | | 1 or less | | >=8 | 6 | | 191.3 | 7 | | 251.1 | 9 | | 324.8 | 7 | | 258.1 | 9 | | 268.7 | 10 | | 308.5 | 16 | | 261.4 |
|  |  | 2 | | >1 to 10 | | >8-80 | 39 | | 1243.5 | 39 | | 1399.2 | 35 | | 1263.0 | 32 | | 1180.1 | 29 | | 865.9 | 29 | | 894.6 | 36 | | 588.2 |
|  |  | 3 | | >10 to 21 | | >80-168 | 15 | | 478.3 | 20 | | 717.5 | 19 | | 685.6 | 21 | | 774.4 | 23 | | 686.8 | 22 | | 678.7 | 14 | | 228.8 |
|  | Increasing risk | 4 | | >21-35 | | >168-280 | 11 | | 350.7 | 14 | | 502.3 | 14 | | 505.2 | 16 | | 590.0 | 14 | | 418.0 | 14 | | 431.9 | 9 | | 147.1 |
|  |  | 5 | | >35-50 | | >280-400 | 4 | | 127.5 | 4 | | 143.5 | 4 | | 144.3 | 6 | | 221.3 | 7 | | 209.0 | 7 | | 215.9 | 3 | | 49.0 |
|  | Higher risk | 6 | | >50 | | >400 | 5 | | 159.4 | 4 | | 143.5 | 6 | | 216.5 | 6 | | 221.3 | 7 | | 209.0 | 5 | | 154.2 | 2 | | 32.7 |
| **Female** | Abstainer |  | | 0 | | 0 | 24 | | 730.5 | 20 | | 721.4 | 17 | | 615.6 | 16 | | 595.2 | 19 | | 579.5 | 22 | | 541.6 | 35 | | 786.6 |
|  | Lower risk | 1 | | 1 or less | | >=8 | 7 | | 213.0 | 15 | | 541.1 | 14 | | 507.0 | 17 | | 632.4 | 16 | | 488.0 | 22 | | 541.6 | 24 | | 539.4 |
|  |  | 2 | | >1-7 | | >8-56 | 39 | | 1187.0 | 35 | | 1262.5 | 34 | | 1231.2 | 32 | | 1190.4 | 30 | | 914.9 | 28 | | 689.3 | 23 | | 516.9 |
|  |  | 3 | | >7-14 | | >56-112 | 14 | | 426.1 | 15 | | 541.1 | 17 | | 615.6 | 12 | | 446.4 | 14 | | 427.0 | 13 | | 320.1 | 9 | | 202.3 |
|  | Increasing risk | 4 | | >14-21 | | >112-168 | 7 | | 213.0 | 6 | | 216.4 | 7 | | 253.5 | 9 | | 334.8 | 7 | | 213.5 | 6 | | 147.7 | 3 | | 67.4 |
|  |  | 5 | | >21-35 | | >168-280 | 5 | | 152.2 | 6 | | 216.4 | 7 | | 253.5 | 10 | | 372.0 | 10 | | 305.0 | 7 | | 172.3 | 3 | | 67.4 |
|  | Higher risk | 6 | | >35 | | >280 | 5 | | 152.2 | 4 | | 144.3 | 5 | | 181.1 | 5 | | 186.0 | 5 | | 152.5 | 3 | | 73.9 | 1 | | 22.5 |

*See Box 1. Population, n (1000s) by age and sex are from Office for National Statistics 2012 [32] and consumption percentages (published as integers) by age and sex from the Health Survey for England 2012.[9]
